# Sputum from patients with primary ciliary dyskinesia contains high numbers of dysfunctional neutrophils and inhibits efferocytosis

**DOI:** 10.1186/s12931-022-02280-7

**Published:** 2022-12-17

**Authors:** Marfa Blanter, Maaike Cockx, Liesel Wittebols, Sara Abouelasrar Salama, Mirre De Bondt, Nele Berghmans, Noëmie Pörtner, Lotte Vanbrabant, Natalie Lorent, Mieke Gouwy, Mieke Boon, Sofie Struyf

**Affiliations:** 1 Laboratory of Molecular Immunology, Department of Microbiology, Immunology and Transplantation, Rega Institute for Medical Research, University of Leuven, 3000 Leuven, Belgium; 2 Pneumology and Cystic Fibrosis Unit, Department of Pneumology, University Hospitals Leuven, 3000 Leuven, Belgium; 3 Pediatric Pneumology and Cystic Fibrosis Unit, Department of Pediatrics, University Hospitals Leuven, 3000 Leuven, Belgium

**Keywords:** Primary ciliary dyskinesia, Neutrophil, Airway, Inflammation, Bronchiectasis, Sputum, Neutrophil elastase, Efferocytosis

## Abstract

**Background:**

Primary ciliary dyskinesia (PCD) is a genetic disorder characterized by recurrent airway infection and inflammation. There is no cure for PCD and to date there are no specific treatments available. Neutrophils are a crucial part of the immune system and are known to be dysfunctional in many inflammatory diseases. So far, the role of the neutrophils in PCD airways is largely unknown. The purpose of this study was to investigate the phenotype and function of airway neutrophils in PCD, and compare them to blood neutrophils.

**Methods:**

Paired peripheral blood and spontaneously expectorated sputum samples from patients with PCD (n = 32) and a control group of patients with non-PCD, non-cystic fibrosis bronchiectasis (n = 5) were collected. The expression of neutrophil-specific surface receptors was determined by flow cytometry. Neutrophil function was assessed by measuring the extent of actin polymerization, production of reactive oxygen species (ROS) and release of neutrophil extracellular traps (NETs) in response to activating stimuli.

**Results:**

Sputum neutrophils displayed a highly activated phenotype and were unresponsive to stimuli that would normally induce ROS production, actin polymerization and the expulsion of NETs. In addition, PCD sputum displayed high activity of neutrophil elastase, and impaired the efferocytosis by healthy donor macrophages.

**Conclusions:**

Sputum neutrophils in PCD are dysfunctional and likely contribute to ongoing inflammation in PCD airways. Further research should focus on anti-inflammatory therapies and stimulation of efferocytosis as a strategy to treat PCD.

**Supplementary Information:**

The online version contains supplementary material available at 10.1186/s12931-022-02280-7.

## Background

Primary ciliary dyskinesia (PCD) is a genetic disorder which has an incidence of approximately 1/10,000–1/20,000 [1]. PCD is caused by a defect in the motile cilia: hair-like protrusions at the epithelial cell membrane, mainly present in the respiratory epithelium [1]. The beat-like movement of motile cilia is essential for clearance of mucus and pathogens from the respiratory tract [2]. In patients with PCD, mutations in genes involved in the formation of the cilium result in reduced or absent ciliary motility and subsequent accumulation of pathogens in the airways [2].

Patients with PCD suffer from recurrent upper and lower respiratory tract infections, accompanied by characteristic symptoms, such as wet cough, middle ear infection and nasal obstruction [1, 3]. With time, most patients develop bronchiectasis, which strongly increases the risk of respiratory failure [4].

Currently, there is no cure for PCD. Most available treatments are not evidence-based and aim to improve mucociliary clearance or suppress infection and inflammation [3, 5]. According to the vicious vortex model, as described by Flume et al*.*, bronchiectasis is maintained by a complex and interdependent relationship between epithelial dysfunction, impaired mucociliary clearance, chronic airway infection and persistent neutrophilic inflammation which leads to structural lung damage [6]. So far, none of the treatments used for PCD have been successful in interrupting the vicious vortex, although many novel therapeutic approaches are being explored in non-cystic fibrosis (CF) and CF bronchiectasis [7, 8].

Previous research has shown that the airways of patients with PCD contain elevated numbers of neutrophils and pro-inflammatory cytokines, including the major neutrophil-attracting chemokine CXCL8 [9, 10]. Perhaps counterintuitively, sputum samples from patients with PCD also contained high levels of interleukin 10 (IL-10), a cytokine which normally inhibits the recruitment and activation of neutrophils [9, 11]. Neutrophils are the most abundant leukocyte in the blood and are the first immune cells to arrive at the site of infection. Under normal conditions, once neutrophils arrive at the infection site, they mediate a powerful antibacterial response through phagocytosis, release of proteases and antimicrobial peptides, production of reactive oxygen species (ROS), expulsion of neutrophil extracellular traps (NETs) and stimulation of other immune cells with cytokines and chemokines [12]. In view of the important role of neutrophils in inflammatory diseases [13], a better understanding of their role in PCD is vital for the development of anti-inflammatory therapies.

Several chronic respiratory diseases are characterized by dysfunctional neutrophils in the airways. The most notable examples are cystic fibrosis (CF), non-CF bronchiectasis (a broad group of diseases which encompasses PCD as well) and chronic obstructive pulmonary disorder (COPD). All of these diseases have a high influx of neutrophils that apparently fail to clear bacterial infections [14, 15]. In CF, airway neutrophils have been found to have lower phagocytic capacity and reduced production of ROS [14]. In addition, CF and non-CF bronchiectasis sputum contains high levels of free DNA and pro-inflammatory factors, which can be reduced by treatment with macrolide antibiotics [16, 17]. Similarly, spontaneous NET formation by airway neutrophils in COPD has been reported [18]. Together, these findings suggest that airway neutrophils in chronic lung disorders fail to clear infection and instead damage the host tissue, which is also supported by the fact that neutrophil-targeting therapies lead to improved outcome of lung disease in CF [19]. To date, the involvement of airway neutrophils in the disease progression of PCD specifically has as of yet not been reported.

Recently, our group has demonstrated that peripheral blood neutrophils from patients with PCD exhibit ineffective migration and respond less readily to bacterial stimuli compared to neutrophils from healthy individuals [20, 21]. This may indicate that even before migration to the lung, neutrophils in PCD show signs of dysfunction. However, these findings cannot be directly extrapolated to airway neutrophils, as neutrophils undergo significant changes upon migration from the blood into the tissue [22]. Therefore, more research is needed to determine if the reduced responsiveness extends to the neutrophils infiltrating the airways and whether this is a primary or a secondary effect of the genetic mutation. This research will allow us to better understand the pathogenesis of PCD and hopefully provide targets for the development of new therapies or targeted application of already existing therapies.

In this study, we aimed to characterize the phenotype and function of neutrophils from sputum of patients with PCD and compared them to autologous peripheral blood neutrophils. To investigate whether our findings were unique for PCD or typical for bronchiectasis in general, we included a group of patients with non-CF, non-PCD bronchiectasis (further referred to as bronchiectasis).

## Methods

### Patient samples

Between 2017 and 2022, 32 patients with PCD, diagnosed according to the European guidelines, and 5 non-CF, non-PCD idiopathic bronchiectasis were recruited at the University Hospital of Leuven. Each patient donated at least one spontaneously expectorated sputum sample and 10 ml of blood collected in an EDTA-coated tube (BD Biosciences, Franklin Lakes, NJ, USA), with a total of 54 (PCD) + 5 (non-CF bronchiectasis) samples. Adult healthy controls (n = 6) recruited at the host laboratory provided a blood sample only.

All participants (or their parents) signed an informed consent. The study protocol (S57236[ML11095]) was approved by the ethical committee of KU Leuven/UZ Leuven.

### Neutrophil purification

Peripheral blood neutrophils were purified by EasySep immunomagnetic neutrophil isolation (StemCell Technologies, Vancouver, BC, Canada) according to the manufacturer’s instructions. This method is based on negative selection of neutrophils using magnetic antibody-coated beads directed against lineage markers of non-neutrophil leukocytes and erythrocytes. This method consistently yields neutrophils with > 95% purity [23].

To isolate leukocytes from the sputum samples, the protocol of Weiszhar et al*.* was applied [24]. In short, the sputum sample was mixed with 3 ml phosphate-buffered saline (PBS) containing 0.1% dithiothreitol (DTT). The sample was vortexed for 5 min and subsequently incubated for 20 min on a rotary shaker. The sputum was then filtered through a 40 µm cell strainer (Corning Falcon, Corning, NY, USA) and spun down (10 min, 400*g*, 20 °C). The supernatant was collected and stored at − 80 °C until further analysis. The cells were resuspended in PBS and used for flow cytometry and functional assays.

In view of the short life span of neutrophils [25], all samples were processed within one hour after collection.

### Flow cytometry

To characterize the phenotype of sputum and peripheral blood neutrophils, we performed flow cytometry staining using various surface markers known to be expressed on neutrophils during steady-state or pathology (Additional file 1: Table S1).

All reagents used for flow cytometry are listed in Additional file 1: Table S2. 100,000–300,000 cells or 10–30 µl whole blood were used per sample. All centrifugations in the staining procedure were performed for 2 min at 300*g*. The cells were washed once with PBS and incubated with human Fc receptor blocking agent and live/dead dye for 15 min at room temperature (RT) in the dark. Subsequently, the cells were washed twice with flow cytometry buffer (PBS containing 2% fetal calf serum [FCS; Sigma-Aldrich, St Louis, MO, USA] and 2 mM EDTA) and incubated with antibodies for 25–30 min at 4 °C in the dark. Following incubation, the cells were washed twice with flow cytometry buffer and resuspended in 300 µl fixative (0.4% formaldehyde in PBS) for at least 15 min. For some samples, an additional erythrocyte lysis step was needed; in this case, the samples were incubated for 15 min at RT in 800 µl lysis buffer (BD) after the fixation step. Following the lysis, cells were washed twice with flow cytometry buffer and finally resuspended in 300 µl flow cytometry buffer. Acquisition of the samples was done with an LSRFortessa™ X-20 equipped with DIVA software. Analysis was performed with FlowJo software version 10.7.1.

### Chemiluminescence ROS assay

Purified blood neutrophils or sputum cells were resuspended in RPMI medium without phenol red (Gibco, Waltham, MA, USA) at a concentration of 1.5 × 10^6^ cells/ml or 3 × 10^6^ cells/ml, respectively. Prior to stimulation, the cells were incubated for 10 min at 37 °C in the presence of 50 ng/ml tumor necrosis factor-α (TNF-α; Peprotech, Rocky Hill, NJ, USA). Subsequently, the cells were added to a white clear-bottom 96-well plate (Perkin-Elmer, Waltham, MA, USA) and supplemented with luminol (5 mM; Sigma-Aldrich) and one of the following compounds: lipopolysaccharide (LPS) from *Pseudomonas aeruginosa* (10 µg/ml; Sigma-Aldrich); peptidoglycan (PGN) from *Staphylococcus aureus* (10 µg/ml; Sigma-Aldrich); N-formyl-methionyl-leucyl-phenylalanine (fMLF) (10^–8^ M; Sigma-Aldrich). Phorbol 12-myristate 13-acetate (PMA; 150 ng/ml; Sigma-Aldrich) was used as a positive control. An additional condition without luminol was included to determine the background luminescence. The light signal was measured every minute for a period of 3 h by a Clariostar Monochromator microplate reader (BMG Labtech, Orthenberg, Germany). Analysis was performed by subtracting the background luminescence and determining the maximal luminescence as a measure for maximal ROS production. The maximal ROS production of cells stimulated by one of the compounds was normalized to the ROS production of unstimulated cells.

### NETosis assay

NETosis assay was performed as described by Cockx et al*.* [21]. A black clear-bottom 96-well plate (Greiner Bio-One, Kremsmünster, Austria) was coated with 100 µl poly-L-lysine (100 µg/ml; Sigma-Aldrich) per well and incubated for at least 1 h at RT. Subsequently, the plate was washed twice with 200 µl MQ and air-dried in the sterile environment of a biosafety cabinet. Purified blood neutrophils or sputum cells were resuspended in RPMI medium without phenol red with 50 nM SYTOX Green (Invitrogen, Waltham, MA, USA) at a concentration of 0.5 × 10^6^ cells/ml and added to the plate (90 µl/well). The plate was incubated for 30 min at 37 °C to allow adhesion of the cells to the surface. After 30 min, 10 µl of RPMI medium or PMA (final concentration 150 ng/ml) was added to the plate. The cells were incubated for 3 h at 37 °C, capturing phase-contrast images and measuring green fluorescence every hour using the IncuCyte S3 live cell imaging system (Sartorius, Göttingen, Germany). Green fluorescent area was determined using the IncuCyte analysis software (Sartorius) as described before [21]. PMA-induced NETosis was calculated by subtracting the green fluorescence area at t = 0 h from the green fluorescence area at t = 3 h and normalizing this to the unstimulated condition.

### Actin polymerization assay

Purified blood neutrophils or sputum cells were resuspended in actin polymerization buffer (RPMI-1640 + 1 mg/ml human serum albumin [HSA; Belgian Red Cross]) at a concentration of 1.5 × 10^6^ cells/ml and shortly (30 s) exposed to a chemotactic factor: CXCL8 (1–10 ng/ml; Preprotech) or fMLF (10^–8^–10^–9^ M; Sigma-Aldrich). Subsequently, the cells were placed on ice and incubated with ice-cold BD CytoFix/CytoPerm buffer (BD Biosciences) for 15 min at 4 °C. The cells were then washed once with cold BD Perm/Wash buffer (BD Biosciences) (all centrifugations in this assay were performed for 2 min at 300 g, 4 °C) and incubated with AF555-Phalloidin (diluted 1:100 in BD Perm/Wash buffer; Invitrogen) for 20 min at 4 °C. Next, the cells were washed twice with BD Perm/Wash buffer and resuspended in 200 µl flow cytometry buffer (PBS + 2% FCS + 2 mM EDTA). For sputum cells, an additional staining step with anti-CD16 and anti-CD66b was included to distinguish between neutrophils and other cell types. The cells were incubated for 10 min at 4 °C in flow cytometry buffer to allow blocking of the Fc receptors by FCS. Thereafter, 1 µl of anti-human CD66b-BV421 antibody (BD) and 0.5 µl of anti-human CD16-BUV395 antibody (BD Biosciences) was added. The cells were incubated for 30 min at 4 °C in the dark, whereupon they were washed twice with flow cytometry buffer and resuspended in 200 µl flow cytometry buffer. Samples were kept at 4 °C in the dark until acquisition. Acquisition of the samples was done with an LSRFortessa™ X-20 equipped with DIVA software. Analysis was performed with FlowJo software version 10.7.1.

### Measurement of neutrophil elastase concentration and activity

Neutrophil elastase (NE) concentration was measured using the human neutrophil elastase DuoSet ELISA kit (R&D systems, Minneapolis, MN, USA), according to the manufacturer’s instructions.

Neutrophil elastase activity was measured as described by Cambier et al. [26]. In short, dye-quenched elastin (DQ-elastin, ThermoFisher Scientific; final concentration 15 µg/ml) was added to cell-free sputum supernatant (diluted 1:100 in 0.1 M Tris buffer with pH 8) in a 3:1 ratio in a black 384-well plate. Subsequently, fluorescence was measured over time with a CLARIOstar microplate reader (BMG Labtech, Ortenberg, Germany) for 1 h at 37 °C. A standard series consisting of native human NE (Abcam, Cambridge, United Kingdom) was included. To ensure that the measured elastinolytic activity was specifically caused by NE, a condition was included for each sample in which the specific NE inhibitor sivelestat (100 µg/ml; R&D Systems) was added. NE activity was determined by calculating the slope of the fluorescence increase over time.

### Generation of M2 macrophages

Peripheral blood mononuclear cells (PBMCs) were isolated from the peripheral blood of healthy donors using density-gradient separation as described previously [23]. In short, 10 ml whole blood (diluted 1:1 in PBS) was loaded onto 30 ml Pancoll (1.077 g/ml; PAN-Biotech, Aidenbach, Germany) and centrifuged for 30 min at 400 g without braking. Following the centrifugation, the PBMC-containing band above the Pancoll was collected and washed twice with PBS. The cells were suspended in differentiation/polarization medium (RPMI-1640 + 10% FCS + 50 µg/ml gentamicin [Gibco, Waltham, MA, USA]) and plated in a 48-well plate at a density of 0.3 × 10^6^ cells per well (300 µl/well). Subsequently, the PBMCs were incubated for 3-6 h at 37 °C to allow monocyte adhesion. Following incubation, the medium was removed and the cells were washed once with PBS to eliminate the lymphocytes. Subsequently, differentiation medium with 100 ng/ml macrophage colony-stimulating factor (M-CSF, Peprotech) was added, and the cells were incubated for 5 days at 37 °C. After 5 days, the medium was removed and fresh polarization medium containing 100 ng/ml M-CSF and 20 ng/ml interleukin 4 (IL-4, Peprotech) was added. Finally, the cultures were incubated for an additional 2 days.

### Induction of apoptosis in neutrophils

Purified neutrophils from peripheral blood of healthy donors were suspended in carboxyfluorescein succinimidyl ester (CFSE; 1 µM in PBS; Invitrogen) at a density of 5 × 10^6^ cells/ml and incubated for 10 min at RT. Subsequently, 4 volumes of cold RPMI-1640 + 10% FCS were added, and the cells were incubated for 5 min at 4 °C. The cells were then washed 3 times with RPMI-1640 + 10% FCS, whereupon the pellet was resuspended in staurosporine (1 µg/ml in PBS; Sigma) at a density of 1 × 10^6^ cells/ml. The cells were incubated for 2 h at 37 °C with gentle agitation every 30 min. Following incubation, the cells were washed twice with PBS and resuspended in efferocytosis medium (RPMI-1640 without phenol red + 10% FCS + 50 µg/ml gentamicin) at a concentration of 1.8 × 10^6^ cells/ml.

### Efferocytosis assay

Healthy human monocyte-derived M2 macrophages were stained for 30–45 min with 0.5 µM CellTracker Deep Red (Molecular Probes, Eugene, OR USA), diluted in live cell imaging solution (Molecular Probes). Subsequently, the excess CellTracker was removed, and efferocytosis medium with 40% sputum was added. To test whether specifically NE inhibited efferocytosis, the specific NE inhibitor sivelestat (225 µg/ml) or the vehicle DMSO (1.13%) was added. The cells were incubated for 3 h at 37 °C whereupon 50 µl of apoptotic neutrophils (0.09 × 10^6^ cells) were added. The plate was incubated for 37 °C for 1 h; subsequently, the phase contrast and fluorescence (red and green) were captured using the IncuCyte live-cell imaging system. The percentage of efferocytosing macrophages was determined by manually counting the number of macrophages (red) in direct contact with neutrophils (green) and dividing this number by the total number of macrophages. At least 4 pictures were analyzed per condition.

### Statistical analysis

Descriptive statistics (median, interquartile range) were used to describe patient characteristics. Data in bar graphs are presented as median value with the error bars indicating the interquartile range, and the p values indicated above the bars. Mann–Whitney *U* tests were used to determine differences between patient groups or sample types. Fisher exact test was used for non-numerical variables. Correlations were determined using Spearman’s correlation tests. A p-value below 0.05 was considered significant.

## Results

### Study population

Patient characteristics are listed in *Table **1*.

**Table 1:** Patient characteristics

	PCD (n = 32)	Bronchiectasis (n = 5)	p value
Female/male, no. (%)	17/15 (53/47)	5/0 (100/0)	0.0673
Age in yrs, median (IQR)	19 (15–28.5)	68 (61–80)	0.0001
Ultrastructural defect, n (%)			
Normal ultrastructure	7/32 (22)		
ODA deficiency (complete or partial)	14/32 (44)		
CP deficiency (complete or partial)	8/32 (25)		
IDA deficiency (complete or partial)	5/32 (16)		
Unknown	2/32 (6)		
Genetic defect, n (%)			
DNAH5	7/32 (22)		
DNAH11	6/32 (19)		
CCDC40	4/32 (13)		
DNAAF1	2/32 (6)		
RSPH4	1/32 (3)		
RSPH9	1/32 (3)		
HYDIN	1/32 (3)		
CCDC39	1/32 (3)		
CCDC65	1/32 (3)		
OFD1	1/32 (3)		
DNAL1	1/32 (3)		
Unknown	6/32 (19)		
Clinical and laboratory features			
Bronchiectasis, n (%)	30/32 (94)	5/5 (100)	0.9999
FEV_1_% of predicted, median (IQR)	82 (66–93)	85 (71–99.5)	0.6026
FVC % of predicted, median (IQR)	92 (82–102)	108 (85.5–129.5)	0.2070
CRP in mg/L, median (IQR)	1.25 (0.23–4.13)	3.20 (2.20–4.43)	0.1905
Sputum bacteria, n (%)			
*Haemophilus influenzae*	8/54 (15)	0/5 (0)	
*Staphylococcus aureus*	11/54 (20)	0/5 (0)	
*Pseudomonas aeruginosa*	10/54 (19)	3/5 (60)	
*Streptococcus pneumoniae*	1/54 (2)	0/5 (0)	
*Moraxella catarrhalis*	2/54 (4)	0/5 (0)	
*Achromobacter xylosoxidans*	1/54 (2)	0/5 (0)	
*Acetinobacter* species	1/54 (2)	0/5 (0)	
None	28/54 (52)	2/5 (40)	
Unknown	0/54 (0)	2/5 (40)	
Treatment, n (%)			
Azithromycin maintenance	16/54 (30)	1/5 (20)	
Other oral antibiotics	10/54 (19)	0/5 (0)	
Inhaled antibiotics	3/54 (6)	1/5 (20)	
Inhaled steroids	6/54 (11)	3/5 (60)	
Inhaled bronchodilators	2/54 (4)	1/5 (20)	
Inhaled hypertonic saline	10/54 (19)	3/5 (60)	

*PCD* Primary Ciliary Dyskinesia; *IQR* interquartile range; *ODA* Outer dynein arm; *CP* Central pair; *IDA* Inner dynein arm; *DNAH* Dynein axonemal heavy chain; *CCDC* Coiled-coil domain containing; *DNAAF1* Dynein axonemal assembly factor 1; *RSPH* Radial spoke head component; *HYDIN* Hydrocephalus-inducing protein homolog; *OFD1* Oral-facial digital syndrome 1; *DNAL1* Dynein axonemal light chain 1; *FEV*_*1*_ Forced expiratory volume in 1 s; *FVC* Forced vital capacity; *CRP* C-reactive protein

### Peripheral blood neutrophils generally have the same phenotype in patients and controls

**Fig. 1:**
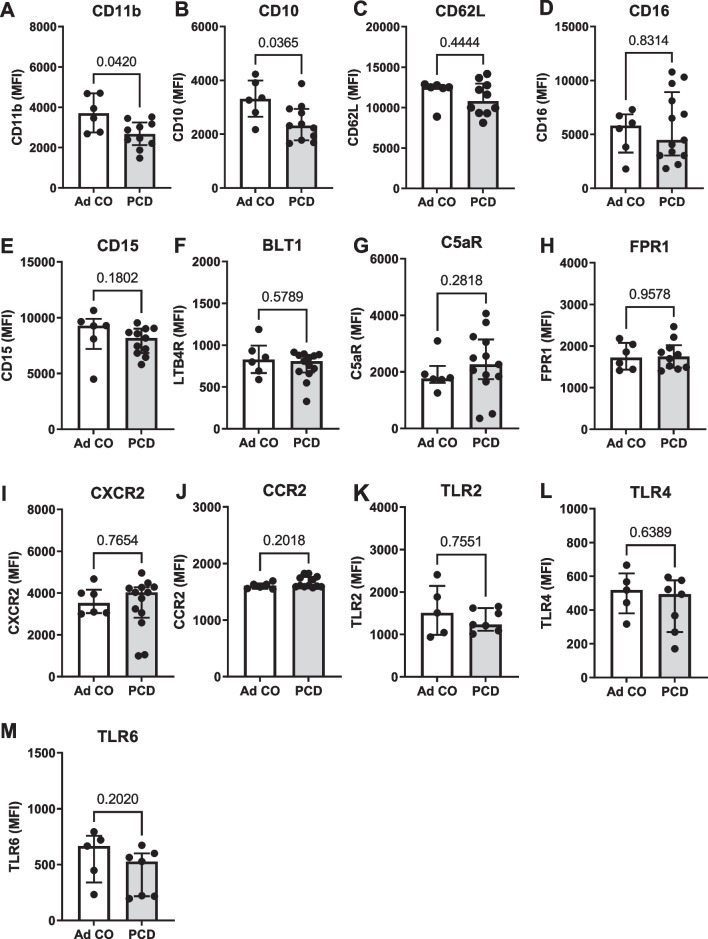
Expression of neutrophil-associated surface markers on healthy and PCD neutrophils. Neutrophils from healthy adult individuals (Ad CO, n = 6) and patients with PCD (PCD, n = 7–13) were isolated from peripheral blood. Using flow cytometry, the expression of **A** CD11b, **B** CD10, **C** CD62L, **D** CD16, **E** CD15, **F** BLT1 (LTB4R), **G** C5aR, **H** FPR1, **I** CXCR2, **J** CCR2, **K** TLR2, **L** TLR4 and **M** TLR6 was determined. Results are presented as median fluorescence intensities (MFI) on the total neutrophil population (gated as CD16^+^CD66b^+^ cells)

First, we investigated the differences in the expression of surface markers on peripheral blood neutrophils between patients with PCD and healthy adult controls (Fig. 1). Peripheral blood neutrophils from patients with PCD had a reduced surface expression of the integrin CD11b (p = 0.0420) and the maturation marker CD10 (p = 0.0365) as compared to healthy controls (Fig. 1A, B). Levels of CD62L, CD16, CD15, BLT1 (LTB4R), C5aR, FPR1, CXCR2, CCR2, TLR2, TLR4 and TLR6 did not differ between PCD and control neutrophils (Fig. 1C–M). CD11c, CXCR4, CCR1, CXCR3, ICAM1 and HLA-DR were not consistently detected (data not shown).

### PCD sputum is mainly composed of neutrophils

**Fig. 2:**
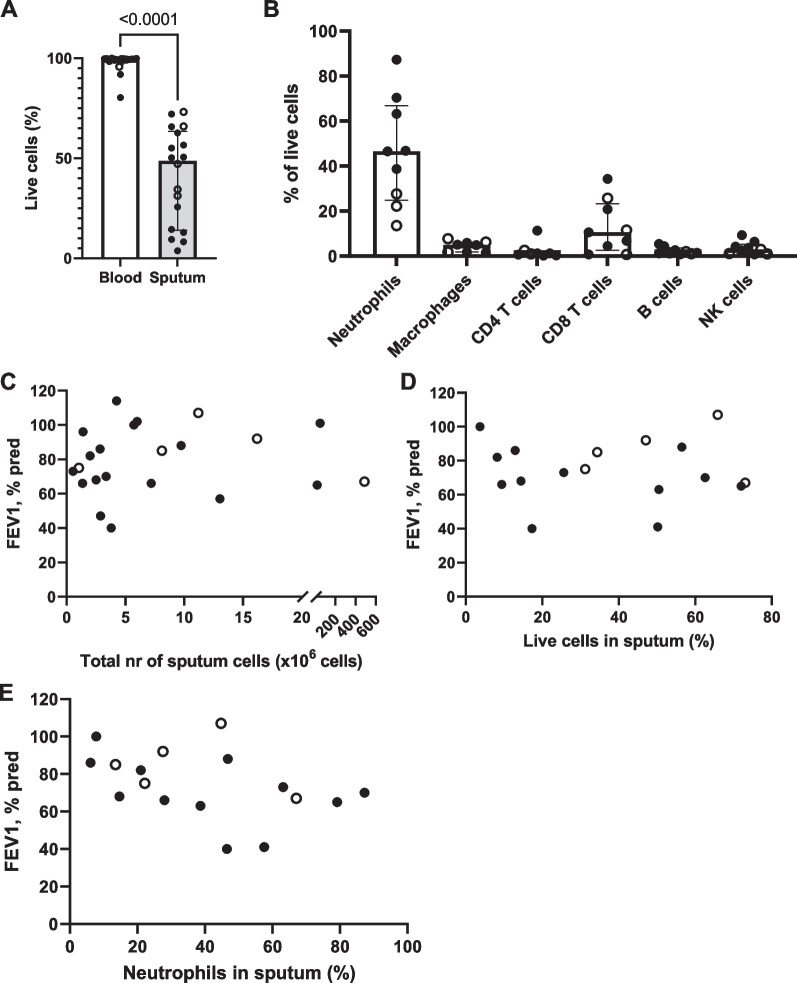
Cell viability and cell composition of sputum cells in PCD and bronchiectasis. **A** Purified peripheral blood neutrophils and purified sputum cells from patients with PCD (black circles, n = 13) and non-CF, non-PCD bronchiectasis (open circles, n = 5) were stained with live/dead dye, and the percentage of live cells was determined by flow cytometry. **B** Purified sputum cells from patients with PCD (black circles, n = 5) and non-CF, non-PCD bronchiectasis (open circles, n = 3) were labeled with antibodies directed against lineage markers (CD16 and CD66b for neutrophils; CD14 and HLA-DR for macrophages; CD4 for helper T lymphocytes; CD8 for cytotoxic T lymphocytes; CD19 for B cells; CD56 for NK cells). Using flow cytometry, the proportion of each leukocyte subpopulation within live cells in the sputum was determined. **C** Association between total number of cells isolated from the sputum samples of patients with PCD (black circles, n = 17) and non-CF, non-PCD bronchiectasis (open circles, n = 5) and the FEV_1_% pred value, p = 0.6238. **D** Association between the percentage of live cells observed in sputum from patients with PCD (black circles, n = 12) and non-CF, non-PCD bronchiectasis (open circles, n = 5) (as determined by the cells being negative for live/dead staining) and the FEV_1_% pred value, p = 0.5920. **E** Association between the percentage of neutrophils in the sputum of patients with PCD (black circles, n = 12) and non-CF, non-PCD bronchiectasis (open circles, n = 5) (defined as the proportion of live cells that was positive for both CD16 and CD66b) and the FEV_1_% pred value, p = 0.0615

Compared to peripheral blood, cells in PCD and bronchiectasis sputum displayed significantly lower viability, although most samples still contained a substantial number of live cells (Fig. 2A). The sputum mainly comprised neutrophils, with lymphocytes, natural killer (NK) cells and macrophages typically being less than 10% of the total number of leukocytes (Fig. 2B). We observed no significant correlation between the lung function (as measured by the forced expiratory volume in 1 s [FEV_1_% predicted]) and the total number of cells isolated per sputum sample (Fig. 2C, *p* = 0.6238), the proportion of live cells (Fig. 2D, *p* = 0.5920) or the proportion of live neutrophils (Fig. 2E, *p* = 0.0615).

### Neutrophils in PCD sputum display a highly activated phenotype

**Fig. 3:**
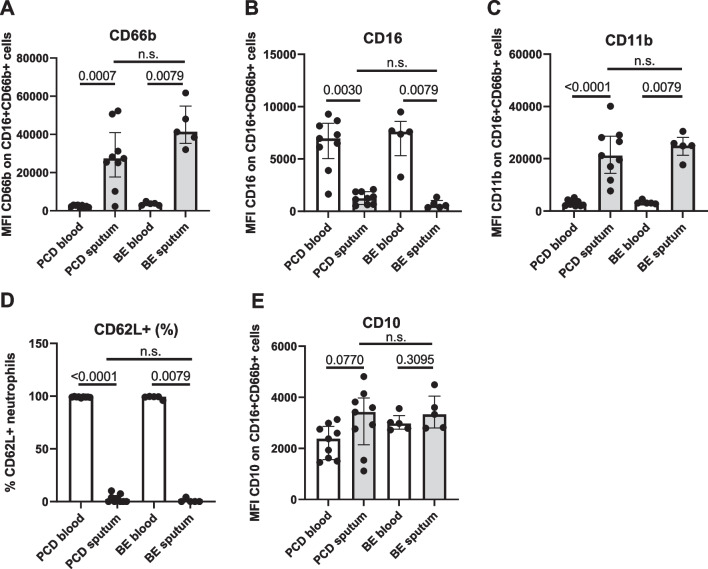
Expression of activation markers on blood and sputum neutrophils in PCD and bronchiectasis. Peripheral blood neutrophils from patients with PCD (PCD blood, n = 9) and non-PCD, non-CF bronchiectasis (BE blood, n = 5) and purified sputum cells from patients with PCD (PCD sputum, n = 9) and non-PCD, non-CF bronchiectasis (BE sputum, n = 5) were labeled with antibodies directed against various activation markers. Using flow cytometry, the levels of **A** CD66b, **B** CD16, **C** CD11b, **D** CD62L, **E** CD10 were determined. Data are shown as **A**–**C**, **E** median fluorescence intensities measured in the neutrophil population (defined as live CD16 + CD66b + cells) or as **D** the percentage of marker-positive cells within the neutrophil population (defined as live CD16 + CD66b + cells)

Both PCD and bronchiectasis sputum neutrophils displayed reduced levels of CD16 (Fig. 3A) and elevated levels of CD66b (Fig. 3B), corresponding to an activated phenotype [27, 28]. Supporting these data, we observed increased levels of the integrin CD11b (Fig. 3C) and a near-complete absence of the selectin CD62L (Fig. 3D) on the cell surface of sputum neutrophils in both PCD and bronchiectasis. The maturation marker CD10 was slightly, but not significantly increased on the surface of PCD sputum neutrophils (Fig. 3E, *p* = 0.0770). CD49d has been described to be increased on neutrophils in several inflammatory diseases, including influenza [29], allergy [30] and juvenile idiopathic arthritis [31]; however, we could not detect expression of CD49d on either the blood or sputum neutrophils of patients with PCD and bronchiectasis (data not shown).

### Sputum neutrophils have a different expression pattern of chemoattractant and Toll-like receptors than blood neutrophils

**Fig. 4:**
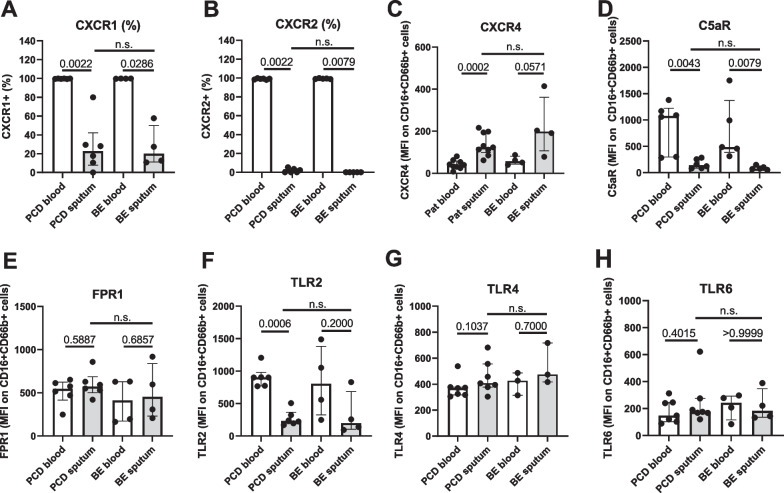
Expression of chemoattractant and Toll-like receptors on the surface of peripheral blood and sputum neutrophils in PCD and bronchiectasis. Peripheral blood neutrophils from patients with PCD (PCD blood, n = 5–7) and non-PCD, non-CF bronchiectasis (BE blood, n = 3–5) and purified sputum cells from patients with PCD (PCD sputum, n = 5–7) and non-PCD, non-CF bronchiectasis (BE sputum, n = 3–5) were labeled with antibodies against various neutrophil receptors. Using flow cytometry, the levels of **A** CXCR1, **B** CXCR2, **C** CXCR4, **D** C5aR, **E** FPR1, **F** TLR2, **G** TLR4 and **H** TLR6 were determined. Data are shown as **A**, **B** percentage of receptor-positive cells within the neutrophil population (defined as live CD16 + CD66b + cells) or as (**C**–**H**) median fluorescence intensities (MFI) measured in the neutrophil population (defined as live CD16 + CD66b + cells)

We detected reduced levels of the chemokine receptors CXCR1 and CXCR2 (Fig. 4A, B) and an increased expression of the chemokine receptor CXCR4 (Fig. 4C) on sputum neutrophils as compared to peripheral blood neutrophils, both in PCD and in bronchiectasis. In addition, reduced levels of the complement receptor C5aR were observed (Fig. 4D). Expression of FPR1 did not differ between blood and sputum cells (Fig. 4E). TLR2, which recognizes i.a. zymosan and peptidoglycan (PGN), was significantly downregulated on sputum cells in both PCD and bronchiectasis (Fig. 4F). Expression of TLR6, which forms a heterocomplex with TLR2, and of the LPS receptor TLR4 was not significantly different between blood and sputum neutrophils (Fig. 4G–H).

### Neutrophils from PCD sputum are not responsive to external stimuli in functional assays

The degree of responsiveness of sputum neutrophils towards bacterial components was assessed in three functional assays: actin polymerization assay, ROS assay and NETosis assay.

**Fig. 5:**
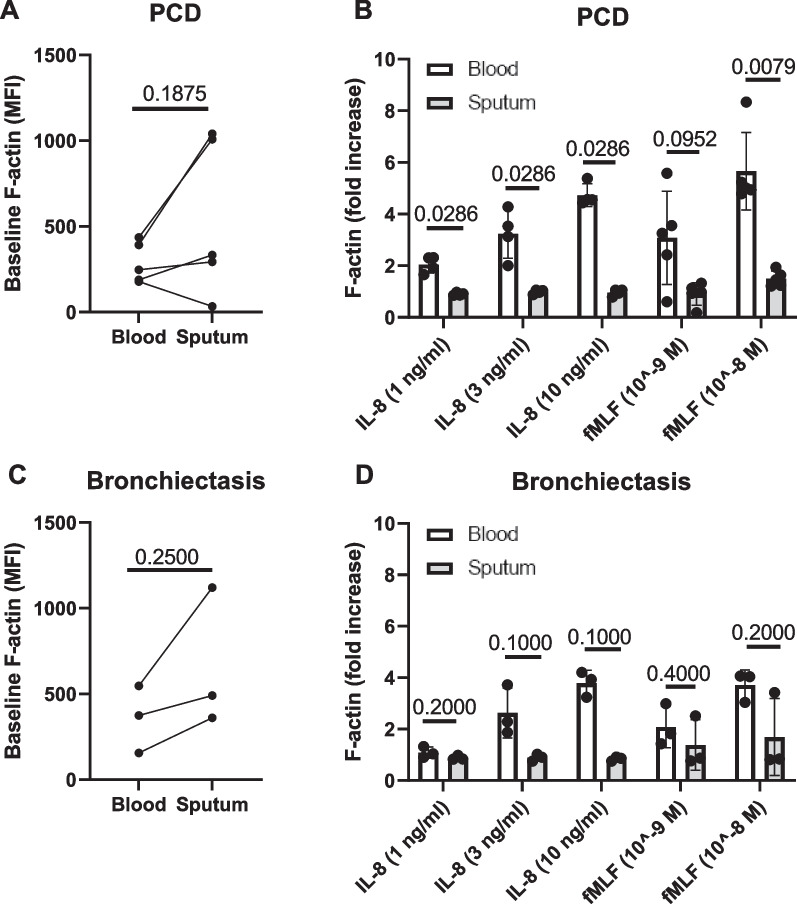
Levels of polymerized actin in peripheral blood and sputum neutrophils in PCD and bronchiectasis. Purified blood neutrophils and sputum cells from patients with **A**, **B**. PCD (n = 4–5) and **C**, **D** non-PCD, non-CF bronchiectasis (n = 3) were exposed for 30 s to a chemotactic stimulus, whereupon the cells were fixed and stained with AF555-phalloidin. Using flow cytometry, the total F-actin content per cell was determined in neutrophils (defined as CD16 + CD66b + cells). Data are shown as **A**, **C** median fluorescence intensities (MFI) of AF555-phalloidin in unstimulated cells or as **B**, **D** the fold increase in F-actin content (MFI) in stimulated cells, relative to the MFI of unstimulated cells

Actin polymerization in neutrophils occurs upon encounter of a chemotactic factor and is the first step in the process of migration [32]. We found that on baseline, PCD sputum neutrophils showed a weak trend towards a higher cellular content of F-actin as compared to blood neutrophils (Fig. 5A). Blood-derived neutrophils readily responded to treatment with CXCL8 and fMLF (Fig. 5B, D); however, stimulation by CXCL8 (IL-8) or fMLF did not evoke an increase in actin polymerization in sputum neutrophils (Fig. 5B). In patients with bronchiectasis, a similar phenotype was found, although sputum neutrophils of one donor did show a prompt reaction towards fMLF (Fig. 5C, D).

**Fig. 6:**
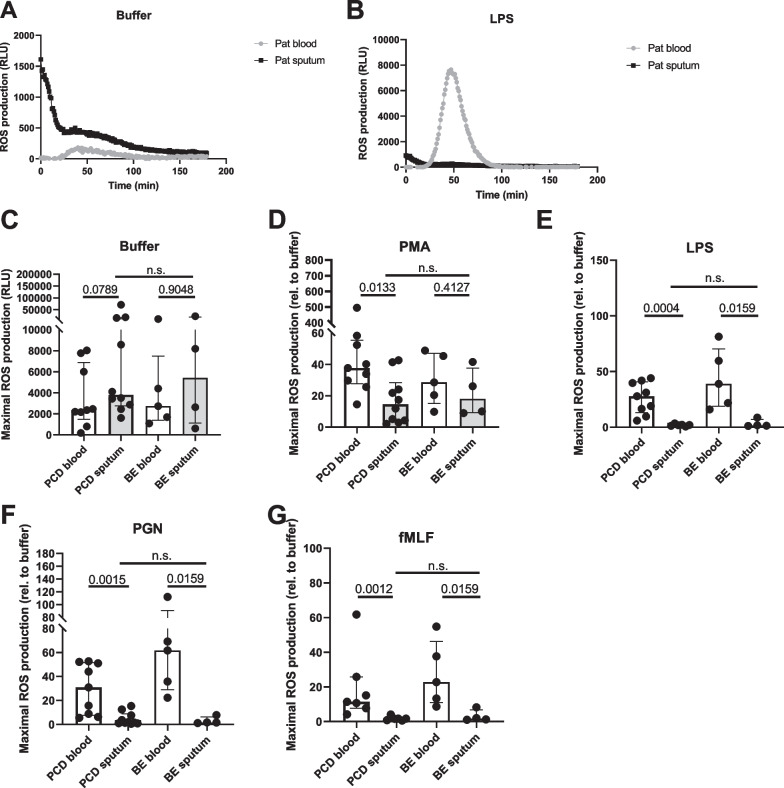
Production of reactive oxygen species by blood and sputum neutrophils in PCD and bronchiectasis. Peripheral blood neutrophils from patients with PCD (PCD blood, n = 6–10) and non-PCD, non-CF bronchiectasis (BE blood, n = 4–5) and purified sputum cells from patients with PCD (PCD sputum, n = 6–10) and non-PCD, non-CF bronchiectasis (BE sputum, n = 4–5) were primed with TNF-α and exposed to PMA or bacterial stimuli. By detection of luminol chemiluminescence (expressed as relative light units, RLU), the production of reactive oxygen species (ROS) was measured. **A** A representative graph showing the pattern of ROS production by blood and sputum neutrophils over time in absence of stimulation. ROS production is represented in RLU. **B** A representative graph showing the pattern of ROS production by blood and sputum neutrophils stimulated with LPS. **C** Spontaneous release of ROS by unstimulated cells. **D**–**G** ROS release in response to stimulation with **D** PMA, **E** LPS, **F** PGN or **G** fMLF is represented here as fold change relative to the unstimulated condition

Peripheral blood neutrophils hardly produced ROS in absence of stimulation (Fig. 6A); stimulation with PMA (a direct activator of protein kinase C [PKC]), LPS, PGN or fMLF evoked a strong ROS response, which peaked around 1.5 h post-stimulation (representative example in Fig. 6B). Spontaneous ROS production by sputum cells was elevated in approximately half of the donors (both PCD and bronchiectasis), and this production decreased gradually over time (Fig. 6A, C). While ROS production in sputum cells was still increased upon stimulation with PMA (Fig. 6D), stimulation with LPS, PGN or fMLF did not result in an enhanced ROS generation (Fig. 6B, E–G). In PCD, PMA-evoked ROS was higher in blood than in sputum cells, whereas in bronchiectasis, there was no difference in response to PMA between blood and sputum cells.

**Fig. 7:**
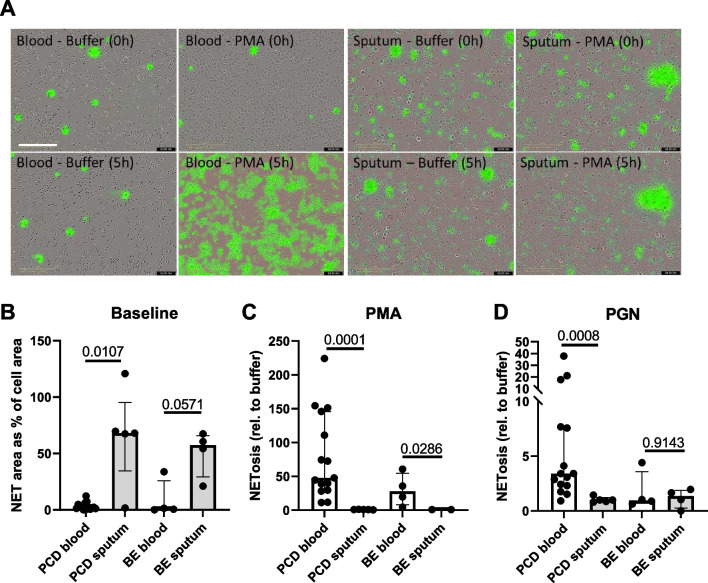
Neutrophil extracellular trap production by peripheral blood and sputum neutrophils in PCD and bronchiectasis. Peripheral blood neutrophils from patients with PCD (PCD blood, n = 15) and non-PCD, non-CF bronchiectasis (BE blood, n = 4) and purified sputum cells from patients with PCD (PCD sputum, n = 5) and non-PCD, non-CF bronchiectasis (BE sputum, n = 4) were exposed to RPMI medium or PMA for 3 h. Using SYTOX Green labeling of extracellular DNA, the degree of NETosis was calculated. **A** Representative microscopy pictures of peripheral blood neutrophils and purified sputum cells at baseline (0 h) and after 3 h of stimulation with PMA. The scalebar is 200 µm. **B** The amount of DNA detectable prior to stimulation. Results are displayed as the green fluorescent area normalized to the cell area. **C** The amount of DNA detectable after exposing the cells for 3 h to PMA. Results are displayed as fold increase in green fluorescent area relative to the unstimulated condition

**Fig. 8:**
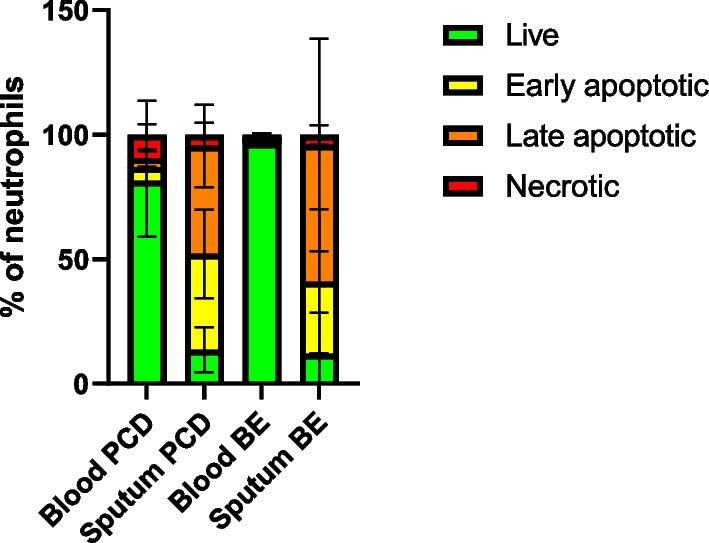
Proportions of apoptotic cells among blood and sputum neutrophils in PCD and bronchiectasis. Purified neutrophils from patients with PCD (PCD blood, n = 4) and non-PCD, non-CF bronchiectasis (BE blood, n = 3) and purified sputum cells from patients with PCD (PCD sputum, n = 4) and non-PCD, non-CF bronchiectasis (BE sputum, n = 3) were stained by a live/dead dye (LD) and Annexin V. Percentages of live (LD^−^AnnV^−^), early apoptotic (LD^−^AnnV^+^), late apoptotic (LD^+^AnnV^+^) and necrotic (LD^+^AnnV^−^) cells are shown

Finally, we performed a NETosis assay, in which we quantified the amount of expulsed DNA by neutrophils in response to PMA (Fig. 7). Peripheral blood neutrophils released relatively little DNA when unstimulated (Fig. 7A, B), but expulsed massive amounts of NETs when exposed to PMA for several hours (Fig. 7A, C). In contrast, many sputum cells already seemed to undergo spontaneous NETosis without stimulation, as witnessed by high amounts of DNA visible under the microscope (Fig. 8A, B). When stimulated with PMA, sputum cells failed to expulse additional DNA into the extracellular space (Fig. 8C). This pattern was seen for cells derived from both PCD and bronchiectasis patients.

To ensure that the lack of response was not caused by the DTT treatment of sputum neutrophils, we isolated peripheral blood neutrophils from healthy donors and exposed them to 0.1% DTT and mechanical disruption, mimicking the processing of sputum cells. Subsequently, we performed actin polymerization, ROS and NETosis assay (Additional file 2: Fig. S1). We found a slight decrease in ROS production in response to PGN (Additional file 2: Fig. S1F), but no complete ablation of the response as seen in the sputum neutrophils.

### Sputum cells are mostly apoptotic

Since we found no responsiveness to external stimuli by sputum neutrophils even in samples where the viability was higher than 50%, we hypothesized that the sputum cells could be in an early stage of apoptosis, which is not detectable by live/dead staining. Therefore, we used Annexin V staining to determine the apoptotic status of the sputum neutrophils. We found that both in PCD and in bronchiectasis, a large proportion of the cells was in early or late stages of apoptosis (Fig. 8).

### PCD sputum inhibits efferocytosis

**Fig. 9:**
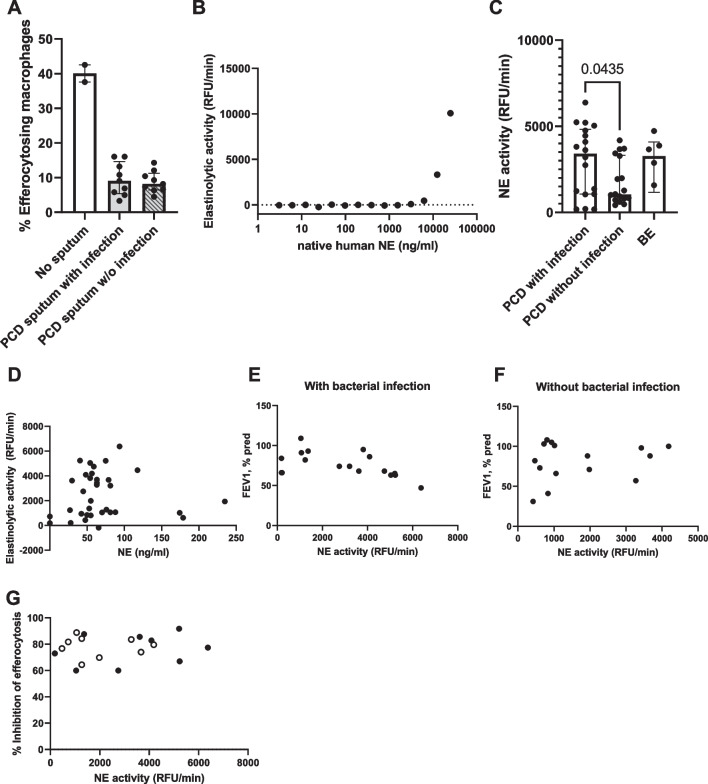
Efferocytosis inhibition and neutrophil elastase activity in PCD sputum. **A** Healthy donor-derived M2 macrophages were exposed for 3 h to sputum from patients with primary ciliary dyskinesia (PCD) with (n = 9) or without (n = 9) positive bacterial sputum culture. Subsequently, apoptotic neutrophils from a healthy donor were added, and the percentage of efferocytosing macrophages was determined. **B** A dilution series of native human neutrophil elastase (NE) was created, and the elastinolytic activity was measured to create a standard curve. Elastinolytic activity is displayed in relative fluorescence units (RFU) per min. **C** Elastinolytic activity was measured in the sputum of patients with PCD with (n = 18) or without (n = 18) positive bacterial sputum culture, and patients with non-PCD, non-CF bronchiectasis with positive bacterial sputum culture (BE, n = 5). **D** The NE immunoreactivity was measured in the sputum of patients with PCD by ELISA, and correlated to the elastinolytic activity in corresponding sputum samples, p = 0.3124. **E** The elastinolytic activity in sputum of PCD patents with positive bacterial sputum culture was measured and correlated to the forced expiratory volume in one second (FEV_1_% pred) as measured on the day that the sputum was collected, p = 0.0422. **F** The elastinolytic activity in sputum of PCD patients without positive bacterial sputum culture was measured and correlated to FEV_1_% pred, p = 0.6830. **G** The elastinolytic activity in sputum of PCD patients with (black circles, n = 9) or without (open circles, n = 9) positive bacterial sputum culture was measured and correlated to the inhibitory effect of sputum on efferocytosis by healthy donor-derived M2 macrophages, p = 0.5505. The inhibitory effect is displayed as % inhibition relative to a control condition without sputum

Based on the high proportion of apoptotic cells we found in the sputum, we hypothesized that a defect in the clearance of these dead cells could be present. One of the most important mechanisms contributing to resolution of inflammation is efferocytosis: the uptake of apoptotic cells by macrophages [33]. To test whether this process was affected in PCD, we exposed healthy human M2 macrophages to PCD sputum for 3 h, whereupon healthy apoptotic neutrophils were added, and the percentage of efferocytosing macrophages was calculated (Fig. 9A). When exposed to PCD sputum, healthy M2 macrophages showed a more than 50% decrease in efferocytosis function, regardless of whether the sputum originated from patients with or without a positive sputum culture.

Previous reports describe several inflammatory agents that can inhibit efferocytosis, among which a prominent candidate is neutrophil elastase (NE). NE is an enzyme released by neutrophils during degranulation and NETosis, and has been linked to both inhibition of efferocytosis and a higher disease severity in CF, COPD and non-CF bronchiectasis [34]. To assess whether NE could be playing a role in PCD, we measured the concentration of NE and its elastinolytic activity in the sputum of patients with PCD. To this end, we first created a standard curve using active native neutrophil elastase (Fig. 9B). This demonstrated that our setup allowed us to accurately detect NE activity in the concentration range between 3 and 25 µg/ml. Subsequently, we measured the elastinolytic activity in sputum from patients with PCD and bronchiectasis (Fig. 9C). We found that both PCD and bronchiectasis sputum contains highly active NE, with significantly higher elastinolytic activity in PCD patients with positive sputum culture, as compared to sputum where no pathogenic bacteria were detected. We observed no correlation between the protein levels and elastinolytic activity (p = 0.3124) (Fig. 9D), suggesting the presence of endogenous NE inhibitors in the sputum. Additionally, we assessed the relation between NE activity and lung function. We observed that NE activity in the sputum was significantly associated with FEV_1_ in patients with an active bacterial infection (p = 0.0380) (Fig. 9E), but not in patients without detectable pathogenic airway bacteria (p = 0.7960) (Fig. 9F).

Finally, we correlated the inhibitory effect of sputum on efferocytosis by healthy donor macrophages with the elastinolytic activity of the corresponding sputum sample (Fig. 9E). We observed no correlation (p = 0.5505), indicating that while sputum strongly inhibits efferocytosis, this inhibition is not caused by neutrophil elastase. Supporting these data, the addition of sivelestat (an NE-specific inhibitor) did not improve efferocytosis in macrophages exposed to PCD sputum (*data not shown*).

## Discussion

Primary ciliary dyskinesia (PCD) is a chronic respiratory disorder characterized by recurrent infections and predominantly neutrophilic inflammation in the airways [1, 9]. In this study, we investigated the phenotype and function of neutrophils in spontaneously expectorated sputum of patients with PCD, including non-PCD, non-CF bronchiectasis as a control group.

We observed that airway neutrophils express a pattern of surface markers that is distinct from peripheral blood neutrophils and is indicative of neutrophil activation and ageing (i.e. CD16^low^, CD66b^high^, CD11b^high^, CD62L^low^ and CXCR4^high^), both in PCD and in bronchiectasis. In addition, airway neutrophils exhibited lower expression of the receptors CXCR1, CXCR2, C5aR and TLR2; expression of FPR1, TLR4 and TLR6 did not differ between blood and airway neutrophils. Changes in some of these markers are not necessarily related to disease, but rather to transmigration of neutrophils from blood to the lung. This phenomenon has been described in detail by Fortunati et al*.*, who discovered that even in healthy individuals, airway neutrophils express higher CD11b and CD66b, and downregulate CD62L and C5aR [22]. Interestingly, airway neutrophils from healthy individuals express higher CXCR1 than blood neutrophils, whereas patients with sarcoidosis exhibit lower CXCR1 expression, similar to the PCD and bronchiectasis patients in our cohort [22]. Loss of CXCR1 has also been reported in CF, which could potentially be explained by the fact that neutrophil elastase (NE) is capable of cleaving CXCR1 on the cell surface [14]. Increased CXCR1 has been observed on peripheral blood neutrophils in patients with COPD [35]; however, we did not find any differences in peripheral blood neutrophil CXCR1 expression between patients with PCD and healthy controls in our cohort.

At baseline, we detected a rather high degree of polarization in airway neutrophils from patients with PCD and bronchiectasis, an increased production of ROS and more spontaneous expulsion of DNA compared to their blood counterparts. The increased ROS and NET production in absence of stimulation supports the notion that the neutrophils present in chronic lung disease are hyperactive and damage the host tissue even in the absence of infection [36]. In contrast, when stimulated with chemoattractants or bacterial components, airway neutrophils failed to increase their polarization and release of ROS and NETs. In view of the low CXCR1 and CXCR2 expression on the surface of sputum neutrophils, it was not surprising that they did not react to CXCL8; however, receptors for LPS and fMLF (TLR4 and FPR1, respectively) were not downregulated on sputum neutrophils. Therefore, it is likely that either the sputum neutrophil receptors or the signaling machinery downstream are not functional. This finding could have implications for the clearance of infection from the lungs.

Having found no responsiveness of airway neutrophils to bacterial stimuli, we measured the extent of apoptosis in neutrophils. We observed that the majority of the neutrophils present in the sputum were in early or late stages of apoptosis, likely explaining their unresponsiveness to stimulation. High numbers of apoptotic neutrophils in the airways is a phenomenon that is typical for chronic airway diseases but not for homeostatic conditions or acute disease [37]. Therefore, we believe that even in the absence of (bacterial) infection, chronic inflammation in PCD is maintained by defective clearance of dead neutrophils, as referred to in the vicious vortex model by Flume et al*.* [6], and that this is not specific for PCD.

Apoptotic neutrophils are normally cleared by macrophages in a process called efferocytosis [33]. It is plausible that at least part of the dead cells and cell debris is removed by mucociliary clearance, suggesting that patients with PCD may initially have a higher load of apoptotic cells in the airways following infection, as compared to otherwise healthy individuals with an acute infection. A similar phenomenon is likely to be present in patients with other chronic airway diseases, as bronchiectasis is also associated with secondary impaired mucociliary clearance in the affected regions [36]. Apart from clearing apoptotic neutrophils, cilia could also be involved in removing NETosis components, including neutrophil elastase (NE) and histones. NE is known to inhibit efferocytosis by cleaving phosphatidylserine receptors that are employed by macrophages to recognize apoptotic cells [38]. This mechanism has been well described in CF, COPD and bronchiectasis, where higher levels of NE correlate with increased disease severity [34, 38]. Free histones have also been reported to inhibit efferocytosis, as well as to stimulate neutrophil activation, contributing to the continuous cycle of inflammation [39, 40].

To investigate whether efferocytosis could be affected in PCD, we incubated healthy donor-derived macrophages with sputum and measured the effect on efferocytosis. We observed that PCD sputum consistently reduced efferocytosis by 50–90%, regardless of whether the sputum donor had detectable bacteria in the sputum culture or not. To assess whether this inhibitory effect could be mediated by NE, we measured its concentration and activity in PCD sputum. We detected high NE activity in the sputum of both PCD and bronchiectasis patients, with higher NE activity in samples from patients with positive bacterial sputum culture. This is in line with the results of Ratjen et al*.* who found that NE activity is increased in sputum of pediatric PCD patients with acute exacerbations [41]. It should be noted, however, that a negative sputum culture does not necessarily prove absence of bacterial infection, possibly explaining the broad spread of data in the group of PCD patients without detectable bacteria in the sputum. In the group of PCD patients with positive sputum culture, higher NE activity also correlated with reduced FEV_1_. Of note, we found no correlation between NE activity and NE protein levels, supporting the notion that the balance between NE and native NE inhibitors may vary strongly between the patients, as described by Oriano et al. [42].

We observed no link between NE activity in the sputum and its inhibitory effect on efferocytosis, suggesting that the inhibition is mediated by a different compound. Potential candidates are extracellular histones [39] and TNF-α [43], although it is also possible that the efferocytosis is inhibited as a yet unknown side effect of a different molecule. Importantly, NE has been reported to have various pro-inflammatory properties not related to efferocytosis [34]. Therefore, it is likely that NE activity and the inhibition of efferocytosis have an additive effect that aggravates the inflammation in PCD.

**Fig. 10:**
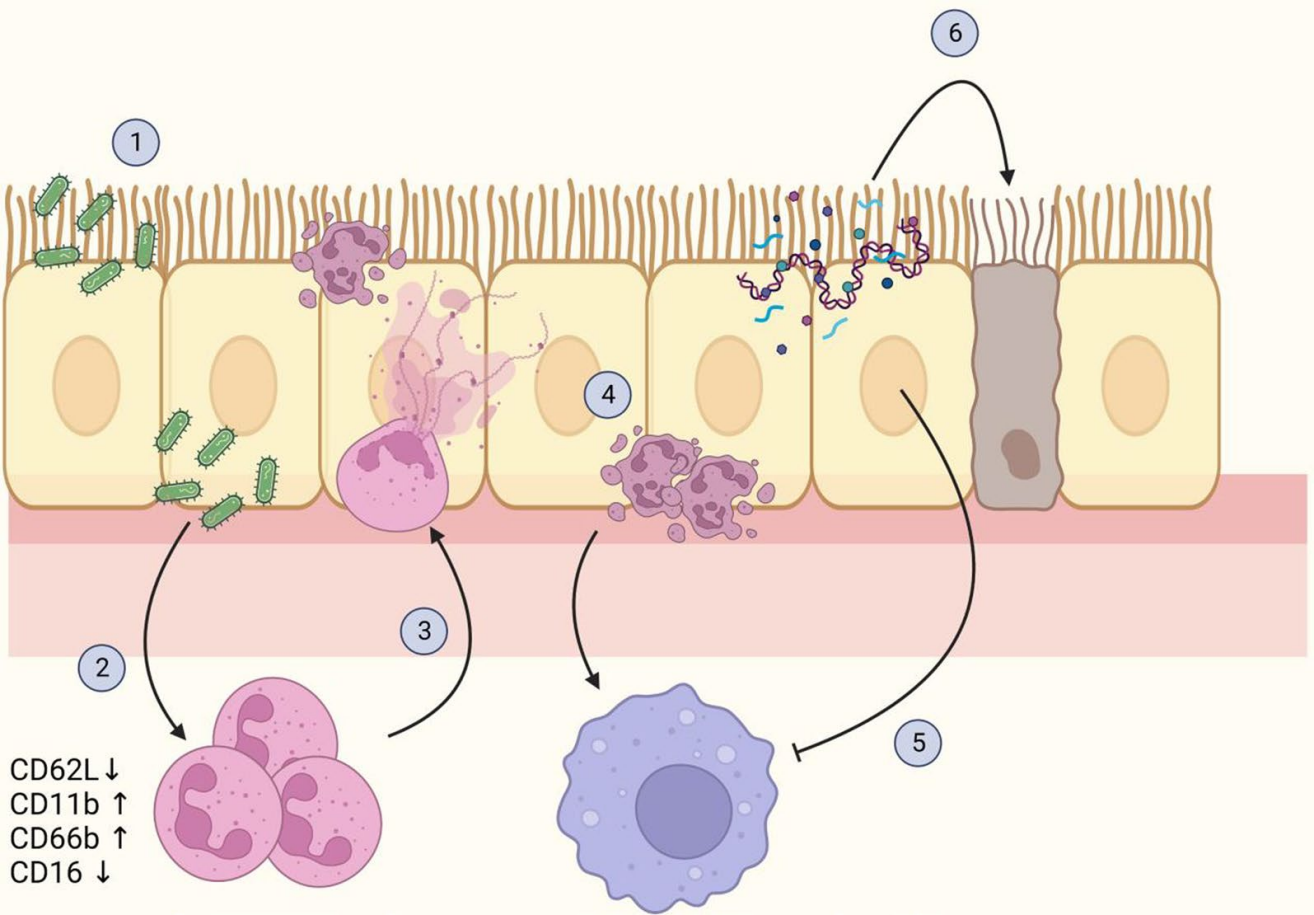
The proposed mechanism of sustained inflammation in PCD. **1** Due to ciliary immotility in PCD, the mucus is not cleared efficiently, leading to increased numbers of pathogens remaining in the airway and establishing an infection. **2** The trapped airway pathogens trigger an acute inflammatory reaction, attracting neutrophils to the airways. Upon transmigration from the blood, the neutrophils become activated, a process accompanied by a change in surface marker expression, including loss of CD62L and CD16, and an increase in CD11b and CD66b. **3** Once in the airways, neutrophils employ different strategies to combat the pathogens, including the release of enzymes (e.g. myeloperoxidase and neutrophil elastase) and neutrophil extracellular traps (NETs). Part of the neutrophils will die through NETosis, whereas the rest of the neutrophils will go in apoptosis. **4** Due to defective mucociliary clearance, excessive numbers of apoptotic cells, as well as DNA and neutrophil-derived enzymes, remain in the airways. The apoptotic neutrophils attract pro-resolving macrophages to clear the dead cells and initiate the resolution of inflammation. **5** Proteases remaining in the airways inhibit the clearance of apoptotic cells by macrophages through receptor cleavage. **6** Neutrophil-derived molecules damage airway cells, aggravating the inflammation and facilitating secondary infection. Created with www.BioRender.com

Together, our findings indicate that the disease phenotype in PCD is mostly maintained by reduced clearance of dead cells and defective resolution of inflammation (Fig. 10). Therefore, we believe that further research into PCD should focus mostly on the resolution of inflammation and tissue healing. For example, treatment with granulocyte–macrophage colony-stimulating factor (GM-CSF) has been shown to enhance clearance of apoptotic neutrophils in mice, and inhaled GM-CSF is well-tolerated by humans as well [44, 45]. Similarly, macrolide antibiotics have been reported to decrease NETosis and improve efferocytosis function in alveolar macrophages [17, 46]. The beneficial effect of azithromycin maintenance therapy in PCD has recently been demonstrated [47]. Therapies promoting resolution of inflammation will likely have to be combined with anti-inflammatory medication, as neutrophils in chronic airway disease may be undergoing immunometabolic reprogramming which includes increased release of NETs and cytokines, and reduced bacterial killing [7]. Clinical trials that aim to inhibit neutrophil activation in PCD are currently ongoing (e.g. NCT04594369).

It is important to note that our study has several limitations. Firstly, our bronchiectasis patient group was limited in size, which could have led to false negative results in some of the statistical tests. Secondly, as we focused specifically on neutrophils, the flow cytometry acquisition parameters were set in a way that the forward and side scatter were best suited for neutrophil detection. Alveolar macrophages and epithelial cells are substantially larger in size than neutrophils, and could therefore fall outside the detection gate. Consequently, the results in Fig. 2 could be skewed. Thus, Noone et al*.* [48] report that approximately 95% of lung leukocytes during steady state are alveolar macrophages. Finally, we studied spontaneously expectorated sputum, which likely contains neutrophils that are at the end of their lifespan and are not necessarily representative for the cells that have recently migrated in the airways. A more representative population would be cells derived from bronchoalveolar lavage, but due to ethical concerns, this was not possible in our study.

## Conclusions

We found that PCD airways contain high numbers of dysfunctional neutrophils and high NE activity, which indicates ongoing inflammation and may result in defective clearance of apoptotic cells, contributing to chronic inflammatory airway disease. To our knowledge, this is the first study characterizing the phenotype and function of airway neutrophils in PCD specifically, as well as linking this to the clearance of apoptotic cells in the airways. We believe that further research into the resolution of inflammation in PCD would be beneficial for better understanding of the disease and development of new treatments or evidence-based application of existing treatments.

## Supplementary Information

**Additional file 1: Table S1.** Surface markers on neutrophils investigated in this study. **Table S2.** Reagents used for flow cytometry.

**Additional file 2: Figure S1.** Effect of DTT treatment on the function of neutrophils.

## Data Availability

The datasets used and analyzed in the current study are available from the corresponding author on reasonable request.

## Abbreviations

Ad CO: Healthy adult control
AnnV: Annexin V
BE: Non-PCD, non-CF bronchiectasis
C5aR: Complement component 5a receptor
CCR: C–C chemokine receptor
CD: Cluster of differentiation
CF: Cystic fibrosis
COPD: Chronic obstructive pulmonary disorder
CXCL: C–X–C motif ligand
CXCR: C–X–C chemokine receptor
DTT: Dithiothreitol
EDTA: Ethylene diamine tetraacetic acid
F-actin: Polymerized (filamentous) actin
FCS: Fetal calf serum
FEV_1_: Forced expiratory volume in 1 s
FPR1: Formyl peptide receptor 1
fMLF: N-formyl methionine leucyl phenylalanine
GM-CSF: Granulocyte–macrophage colony-stimulating factor
HLA-DR: Human leukocyte antigen-DR isotype
ICAM1: Intercellular adhesion molecule 1
IL: Interleukin
LD: Live/dead dye
LPS: Lipopolysaccharide
LTB4R: Leukotriene B4 receptor
M-CSF: Macrophage colony-stimulating factor
MFI: Median fluorescence intensity
NE: Neutrophil elastase
NET: Neutrophil extracellular trap
NK: Natural killer
PBMC: Peripheral blood mononuclear cells
PBS: Phosphate-buffered saline
PCD: Primary ciliary dyskinesia
PGN: Peptidoglycan
PMA: Phorbol 12-myristate 13-acetate
ROS: Reactive oxygen species
RPMI: Roswell Park Memorial Institute
RT: Room temperature
TLR: Toll-like receptor
TNF-α: Tumor necrosis factor-α
