# Supplementary material for: Sputum from patients with primary ciliary dyskinesia contains high numbers of dysfunctional neutrophils and inhibits efferocytosis
Source: Respir Res. 2022 Dec 17;23:359. doi: 10.1186/s12931-022-02280-7 (PMC9758951; doi:10.1186/s12931-022-02280-7)
Supplement: Supplementary file 1 — Additional file 1: Table S1. Surface markers on neutrophils investigated in this study. Table S2. Reagents used for flow cytometry. [file 12931_2022_2280_MOESM1_ESM.docx]

**Supplementary material**

**Table S1. Surface markers on neutrophils investigated in this study.**

| **Surface marker** | **Full name** | **Function** | **Reference** |
| --- | --- | --- | --- |
| C5aR | Activated complement component 5 receptor | Recognition of the complement factor C5a; neutrophil chemotaxis  Expression reduced on neutrophils from patients with severe infection and lung-infiltrated neutrophils | (49) |
| CCR1 | CC chemokine receptor 1 | Recognition of CC chemokines; leukocyte chemotaxis  Expression increased on neutrophils from patients with acute respiratory distress syndrome | (49, 50) |
| CCR2 | CC chemokine receptor 2 | Recognition of CC chemokines; leukocyte chemotaxis  Expression increased during severe inflammation | (49, 51) |
| CD10 | Membrane metalloendopeptidase | Maturation marker; cleaves proinflammatory, vasoactive and neuropeptides | (52) |
| CD11b | Integrin αM | Neutrophil adhesion and transmigration; phagocytosis; intracellular signaling | (53) |
| CD11c | Integrin αX | Leukocyte adhesion  Expression increased on neutrophils from patients with sepsis | (54) |
| CD15 | Sialyl Lewis^x^ | Selectin ligand; neutrophil adhesion | (55) |
| CD16 | Fcγ receptor IIIA | Recognition of immune complexes | (56) |
| CD62L | L-selectin | Neutrophil adhesion and intracellular signaling | (57) |
| CXCR1 | CXC chemokine receptor 1 | Recognition of the chemokines CXCL6 and CXCL8; neutrophil chemotaxis  Expression reduced on neutrophils from patients with asthma and COPD | (49) |
| CXCR2 | CXC chemokine receptor 2 | Recognition of the chemokines CXCL1, CXCL2, CXCL3, CXCL5, CXCL6, CXCL7, CXCL8; neutrophil chemotaxis  Expression reduced on neutrophils from patients with asthma and COPD | (49) |
| CXCR3 | CXC chemokine receptor 3 | Recognition of CXCL4, CXCL4L1, CXCL9, CXCL10, CXCL11; leukocyte chemotaxis  Expression increased on neutrophils from patients with chronic lung inflammation | (49, 58) |
| CXCR4 | CXC chemokine receptor 4 | Recognition of CXCL12; leukocyte chemotaxis  Expression increased on neutrophils from patients with chronic lung inflammation | (49, 59) |
| FPR1 | Formyl peptide receptor 1 | Recognition of several formyl and non-formyl peptides, including the bacterial product fMLF; leukocyte chemotaxis; neutrophil respiratory burst and degranulation | (49, 60) |
| HLA-DR | Human leukocyte antigen -DR isotype | Antigen presentation  Upregulated on neutrophils in certain pro-inflammatory environments | (61) |
| ICAM1 | Intercellular adhesion molecule 1 | Expression increased on reverse-transmigrated neutrophils | (62) |
| BLT1 | Leukotriene B_4_ receptor | Recognition of the chemotactic lipid LTB_4_; leukocyte chemotaxis  Expression reduced on neutrophils from trauma patients | (49, 63) |
| TLR2 | Toll-like receptor 2 | Recognition of cell wall components of gram-positive bacteria (including peptidoglycan), mycoplasma, mycobacteria and yeast  Activation of the innate immune response | (64) |
| TLR4 | Toll-like receptor 4 | Recognition of lipopolysaccharide (component of gram-negative bacteria)  Activation of the innate immune response | (64) |
| TLR6 | Toll-like receptor 6 | Recognition of bacterial lipopeptides (in combination with TLR2)  Activation of the innate immune response | (64) |

**Table S2. Reagents used for flow cytometry**

| **Reagent** | **Clone** | **Dilution** | **Company** | **Catalogue number** |
| --- | --- | --- | --- | --- |
| Annexin V (BV711) | / | 1:24 | BD Biosciences | 563972 |
| Annexin binding buffer | / | 1:10 in MQ | Invitrogen | 00-0055-56 |
| Anti-BLT1 (BV510) | 203/14F11 | 1:260 | BD Biosciences | 15859389 |
| Anti-CCR1 (AF647) | 53504 | 1:240 | BD Biosciences | 557914 |
| Anti-CCR2 (AF488) | K036C2 | 1:120 | Biolegend | 357226 |
| Anti-CCR3 (AF647) | 5E8 | 1:120 | Biolegend | 310710 |
| Anti-CD10 (BV786) | HI10a | 1:120 | BD Biosciences | 564960 |
| Anti-CD11b (BV510) | ICRF44 | 1:40 | BD Biosciences | 563088 |
| Anti-CD11b (PE-Cy7) | ICRF44 | 1:120 | Invitrogen | 25-0118-42 |
| Anti-CD11c (PerCP-eFluor710) | 3.9 | 1:120 | eBioscience | 46-0116-42 |
| Anti-CD14 (PE-CF594) | MφP9 | 1:120 | BD Biosciences | 562334 |
| Anti-CD15 (BUV395) | HI98 | 1:120 | BD Biosciences | 563872 |
| Anti-CD16 (AF700) | 3G8 | 1:240 | BD Biosciences | 557920 |
| Anti-CD19 (FITC) | HIB19 | 1:120 | Biolegend | 302256 |
| Anti-CD4 (AF700) | SK3 | 1:120 | Invitrogen | 56-0047-42 |
| Anti-CD45 (PerCP) | 2D1 | 1:120 | R&D Systems | FAB1430C |
| Anti-CD49d (BV711) | 9F10 | 1:600 | Biolegend | 304332 |
| Anti-CD56 (BV421) | NCAM16.2 | 1:120 | BD Biosciences | 562751 |
| Anti-C5aR (PerCP-Cy5.5) | S5/1 | 1:48 | Biolegend | 344312 |
| Anti-CD62L (APC) | DREG56 | 1:120 | BD Biosciences | 561916 |
| Anti-CD66b (BV421) | G10F5 | 1:120 | BD Biosciences | 562940 |
| Anti-CD8 (BV786) | RPA-T8 | 1:120 | BD Biosciences | 563823 |
| Anti-CXCR1 (PE) | 5A12 | 1:24 | BD Biosciences | 555940 |
| Anti-CXCR2 (FITC) | 6C6 | 1:12 | BD Biosciences | 551126 |
| Anti-CXCR3 (BUV395) | 1C/CXCR3 | 1:240 | BD Biosciences | 565223 |
| Anti-CXCR4 (BUV395) | 12G5 | 1:120 | BD Biosciences | 563924 |
| Anti-FPR1 (AF647) | 5F1 | 1:24 | BD Biosciences | 565623 |
| Anti-HLA-DR (BV650) | L243 | 1:240 | Biolegend | 307650 |
| Anti-ICAM1 (BV711) | HA58 | 1:120 | BD Biosciences | 564078 |
| Anti-TLR2 (BV421) | 11G7 | 1:120 | BD Biosciences | 565350 |
| Anti-TLR4 (FITC) | HTA125 | 1:24 | Invitrogen | MA5-16208 |
| Anti-TLR6 (PE) | TLR6.127 | 1:24 | Biolegend | 334708 |
| BD Lysis buffer | / | 1:10 in MQ | BD Biosciences | 349202 |
| Brilliant stain buffer | / | / | BD Biosciences | 563794 |
| Fc block | / | 1:60 | Miltenyi Biotec | 130-059-901 |
| FVS620 live/dead dye | / | 1:10,000 | BD Biosciences | 564996 |
| Zombie Aqua live/dead dye | / | 1:1000 | Biolegend | 423101 |

**Abbreviations: CCR – C-C chemokine receptor; CD – Cluster of differentiation; C5aR – Complement component 5a receptor; CXCR – C-X-C chemokine receptor; FPR1 – formyl peptide receptor 1; HLA-DR – Human leukocyte antigen DR isotype; ICAM1 – Intercellular adhesion molecule 1; TLR – Toll-like receptor**

**Figure S1. Effect of DTT treatment on the function of neutrophils.** Peripheral blood neutrophils from healthy donors (n = 3-5) were isolated by immunomagnetic beads in the presence (DTT treatment) or absence (Control) of 0.1% DTT and with application of mechanical disruption. Subsequently, the capacity to (A-C) polarize, (D-F) produce ROS or (G-H) to expulse NETs was assessed in response to (A,D,G) buffer, (B) IL-8 (10 ng/ml), (C) fMLF (10^-8‑^M), (E,H) PMA (150 ng/ml) or (F) PGN (10 µg/ml). Results are displayed as (A-C) median fluorescence intensity of AF555-Phalloidin, (D-F) maximal ROS production in relative light units (RLU) or (G-H) area of DNA as percentage of total cell area.
