# Supplementary figures and images for: Sputum from patients with primary ciliary dyskinesia contains high numbers of dysfunctional neutrophils and inhibits efferocytosis
Source: Respir Res. 2022 Dec 17;23:359. doi: 10.1186/s12931-022-02280-7 (PMC9758951; doi:10.1186/s12931-022-02280-7)

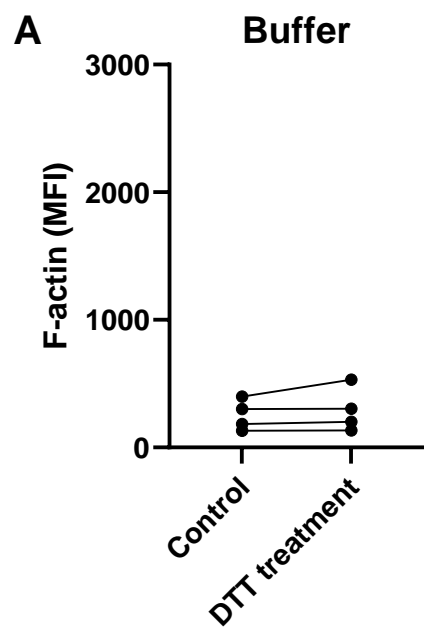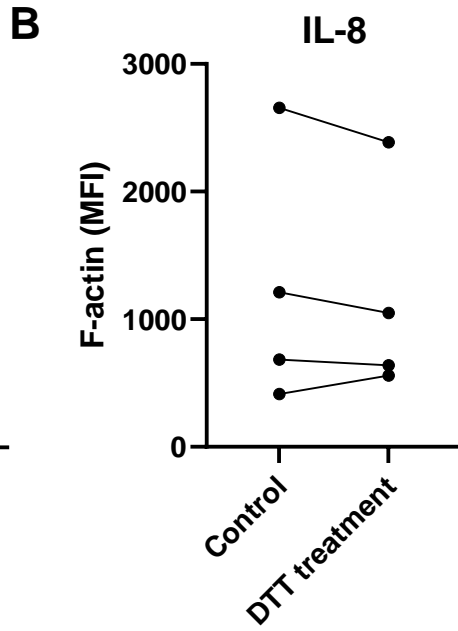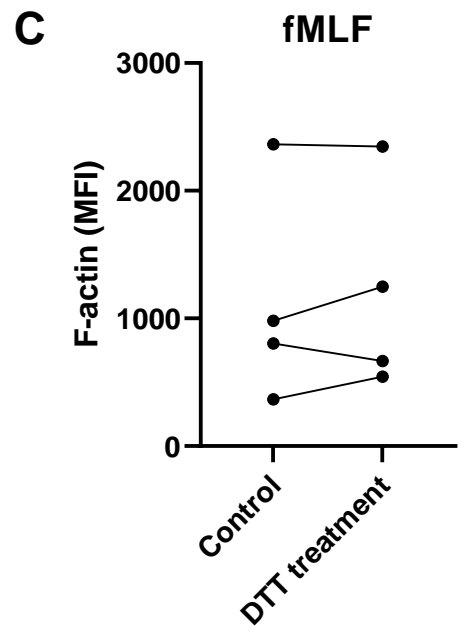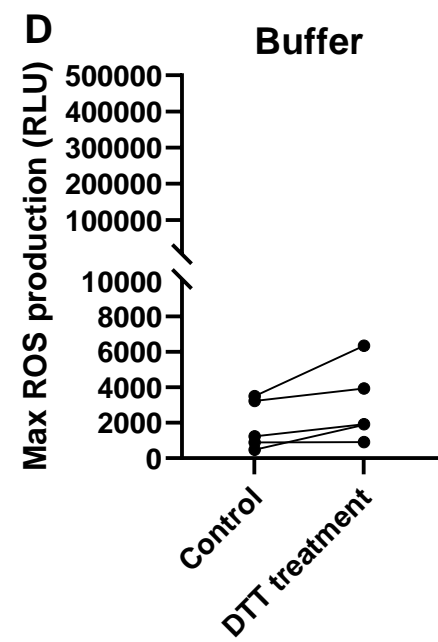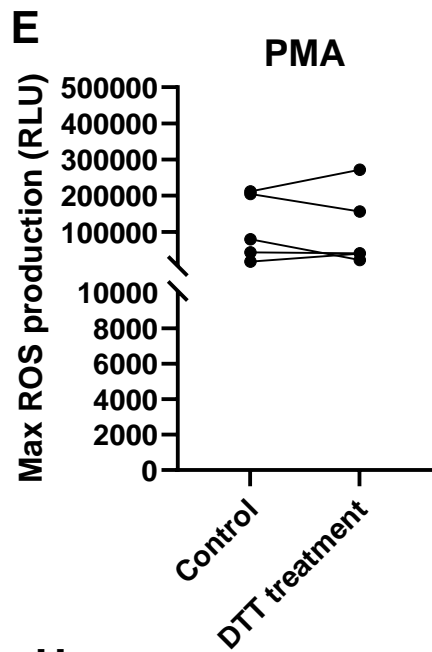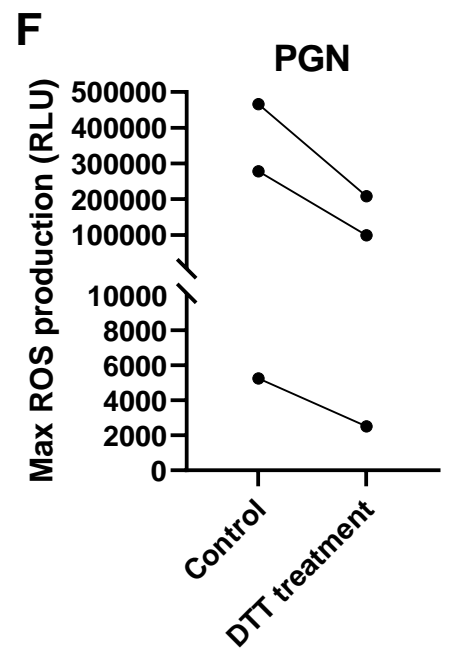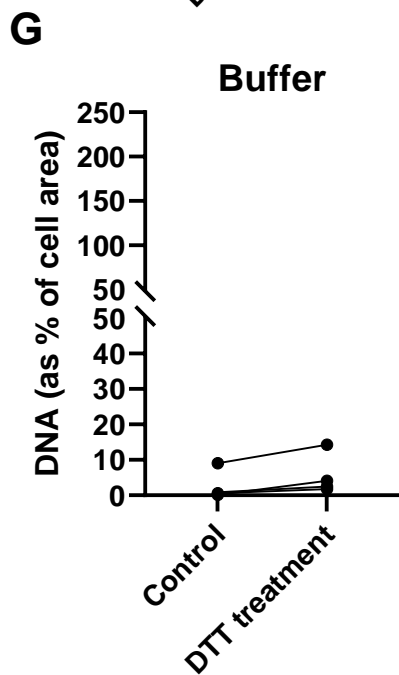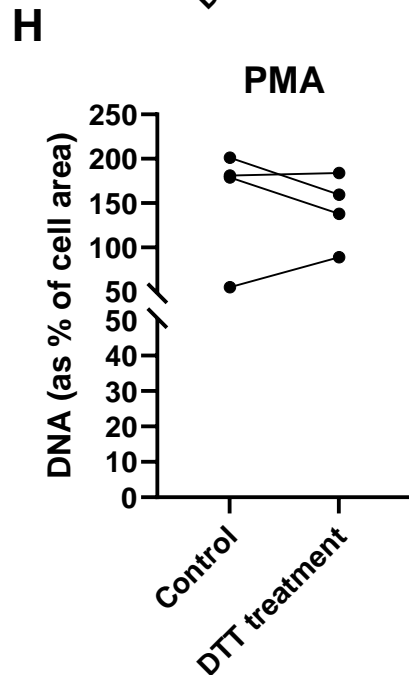

Supplement: Supplementary file 2 — Additional file 2: Figure S1. Effect of DTT treatment on the function of neutrophils. [file 12931_2022_2280_MOESM2_ESM.pdf]
